# Prevalence and incidence of dengue virus and antibody placental transfer during late pregnancy in central Brazil

**DOI:** 10.1186/1471-2334-13-254

**Published:** 2013-05-31

**Authors:** Angela FLT Argolo, Valéria CR Féres, Lucimeire A Silveira, Anna Carolina M Oliveira, Luiz A Pereira, João Bosco Siqueira Júnior, Cynthia Braga, Celina MT Martelli

**Affiliations:** 1 Secretaria de Estado da Saúde de Goiás, Laboratório de Saúde Pública (LACEN-GO), Av. Contorno, 3.556, Jd. Bela Vista, Goiânia, GO, 74.853-120, Brazil; 2 Universidade Federal de Goiás, Instituto de Patologia Tropical e Saúde Publica, Departamento de Saúde Coletiva. Rua Delenda Rezende de Mello, S/N - Sala 405, Setor Universitário, Goiania, GO, 74605-050, Brazil; 3 Universidade Federal de Goiás, Faculdade de Farmácia, Av. Universitária esquina com 1ª Avenida, s/n, St. Universitário, Goiânia, GO, 74.605-220, Brazil; 4 Fundação Oswaldo Cruz, Centro de Pesquisas Aggeu Magalhães. Av Moraes Rego, s/n, Cidade Universitária, Recife, PE, 50000-230, Brazil; 5 Universidade Federal de Pernambuco, Av. Prof. Moraes Rego, s/n - Cidade Universitária, Recife, PE, 50670-420, Brazil

**Keywords:** Dengue, Prevalence, Incidence, Pregnant, Neonate, Vertical transmission

## Abstract

**Background:**

Maternal dengue antibodies are considered to play a significant role in dengue pathogenesis among infants. Determining the transplacental specific antibody transfer is invaluable for establishing the optimal vaccination age among infants in endemic regions.

**Methods:**

We conducted a cross-sectional study among pairs of maternal and corresponding umbilical cord blood samples in public hospitals. The prevalence and incidence of dengue infection were determined in 505 pairs of pregnant women and neonates during a large outbreak (2009–2010) in central Brazil. The women were interviewed at late pregnancy to assess current or past symptoms of dengue. All parturients and their neonates were screened using Dengue IgG Indirect ELISA (Panbio) to assess previous dengue exposure. A semi-quantitative measurement of the IgG antibody expressed by the index ratio was calculated using optical density (OD) values according to the manufacturer’s instructions. The studied population of parturients and their offspring was also screened for recent dengue infection by the Dengue IgM-capture ELISA (Panbio). Those participants with history of fever and two or more symptoms of dengue at least 10 days before the delivery were also tested for the dengue NS1 antigen using the Dengue Early ELISA (Panbio) and RT-PCR.

**Results:**

The mean maternal age was 25.8 (SD = 6.4), and 83.6% of deliveries were between 37 and 41 weeks. Approximately half of the 505 women and neonates were IgG-seropositive, yielding 99.3% co-positive mother-child frequency of antibody transfer (Kappa = 0.96). The incidence of dengue infection was 2.8% (95% CI 1.4–4.4%) among the women considering 14 IgM-positive results and one DENV2 detected by RT-PCR. The dengue NS1 antigen was undetectable in the matched pairs.

**Conclusion:**

This study provides critical data on the prevalence of transplacental transferred maternal-infant anti-dengue antibodies and incidence of infection. The design of future vaccine trials should consider diverse regional epidemiological scenarios.

## Background

Dengue is a vector-borne disease caused by four distinct serotypes (DENV1, 2, 3 and 4), and approximately 3 billion people live or travel in regions infested by *Ae. aegypti* mosquitoes, which are considered the main vector [1]. Dengue has been one of the most important public health issues in tropical and subtropical regions of the world due to the spread of the vector infestation in domestic environments and the potential of DENV infection to cause successive epidemics in highly urbanized settings [2]. DENV infection may progress from asymptomatic to a spectrum of clinical diseases, from mild dengue cases to fatal dengue haemorrhagic fever or dengue shock syndrome (DHF/DSS) [1,3]. Infected individuals may mount a protective immune response for each specific serotype but only short-term heterologous protection against the other serotypes [4]. Consequently, naive populations have a four-fold risk of becoming infected and developing a clinical disease in response to each circulating serotype. Vector control programs have been considered costly, and achieving a sustainable reduction in infestation to low levels is difficult, both of which appear necessary to interrupt the chain of transmission in endemic regions [5]. Potential safe vaccines are under Phase III preventive trials and still need to be tested at a large scale in different endemic settings.

Since the reintroduction of DENV1 in Brazil the late 80’s, the spread of DENV has been reported throughout the country, particularly in the south-eastern and north-eastern regions [6,7]. The isolation of DENV2 was reported in 1990, followed by DENV3 (2000) and DENV4, which were re-introduced in the last two years in most regions [6,8]. More than 1.2 million dengue cases were registered from 2010 to September 2011, in contrast to the 200 thousand registered during 1996–2002. In addition, the proportion of severe clinical forms increased from 0.06% in the 90’s to 0.38% in 2002–2008. These official national data showed growth not only in the incidence but also in the severity of dengue. Although adults are still the most affected population, a steady rise in DENV incidence and hospitalization among children reflects a shift toward a younger age distribution and severity over the past ten years [9]. In part, these current Brazilian epidemiological trends resemble the characteristics of the age distribution of dengue in Southeast Asia, where dengue is a childhood illness. These trends are also in agreement with the theory of dynamic viral infection because the children become the susceptible population after the adults have been infected and immune [10].

Pregnant women and infants are considered a vulnerable group for developing the severe dengue clinical forms according to international and national guidelines. There is no consensus in the literature about the adverse effects of dengue on pregnancy and/or neonates [11,12]. In general, maternal dengue antibodies may play a dual role in the pathogenesis of the dengue disease in the neonates. In the early months of life, transplacental dengue antibodies may protect the infant but may also enhance the risk of developing DHF/DSS due to suboptimal neutralizing antibodies [13,14]. Several studies have suggested that the development of DHF may be related to secondary infections by heterologous serotypes among children and adults or that DHF can occur during the first infection of children who received maternal IgG antibodies [15]. This hypothesis is reinforced by the evidence from Southeast Asia that severe dengue disease in early childhood is mainly due to primary dengue infection [16,17].

This manuscript aims to determine the prevalence of past and recent dengue infections among parturients and their offspring during late pregnancy in central Brazil.

## Methods

### Study setting

We conducted a cross-sectional study among pairs of maternal and corresponding umbilical cord blood samples in two public hospitals located in the city of Goiânia-GO (1.3 million inhabitants) [18] in central Brazil from December 2009 to May 2010. In this setting, the surveillance system registered the first dengue cases in 1994 and detected the co-circulation of three serotypes (DENV1, DENV2 and DENV3) since the year 2002 [19]. During the study period, DENV1 was the predominant virus isolated, replacing the previous predominance of DENV3 circulation between 2002 and 2008 [20]. DENV4 was only detected after the study period (year 2011). In this region, the attack rates were 2.050/100.000 inhabitants (N = 24.603 reported cases) and 3.619/100.000 inhabitants (N = 43.436 cases) for the years 2009 and 2010, respectively [21]. The field work was conducted during a dengue epidemic that occurred in the study setting and in other Brazilian states in 2010 [20].

In this study, 505 parturients were recruited, mean maternal age was 25.8 years (SD = 6.4), and 83.6% of the deliveries were between 37 and 41 weeks. The majority of the participants were unemployed; 60.3% and 24.3% self-referred as biracial or white, respectively. The inclusion criteria were all parturients who were hospitalized during labour and delivery and were residents of the study area, independent of the gestational risk and/or history of infectious or chronic diseases. We also included their neonates.

The parturients were interviewed to collect data on the residence, socio-demographic features, previous dengue episodes and yellow fever vaccination. The main characteristics of the gestational period and delivery were extracted from the medical records. For the neonates, the data were extracted from the official system of live birth registrations (Ministry of Health, Declaration of live births), including the Apgar, birth weight, type of delivery and sex of the neonates. For the women, blood specimens (5 mL) were collected during hospitalization within 48 hrs after delivery. For the neonates, the umbilical cord blood was collected as a routine procedure at the time of delivery. The serum samples were stored until analysis in aliquots at −20°C for serological tests and at −70°C for RT-PCR.

### Laboratory tests

All parturients and their neonates were screened using Dengue IgG Indirect ELISA kits (Inverness Medical Innovations Australia Pty Ltd, Queensland, Australia) to assess the previous dengue exposure. A semi-quantitative measurement of the IgG antibody expressed by the index ratio was calculated using optical density (OD) values according to the manufacturer’s instructions. The studied population of parturients and their offspring were also screened for recent dengue infection by the Dengue IgM-capture ELISA (Inverness Medical Innovations Australia Pty Ltd). Seropositive IgM samples were retested using an in-house IgM capture ELISA test and were considered positive if the ratio of the optical density of the positive to negative controls was at least two [22,23].

Those participants with history of fever and two or more symptoms of dengue at least 10 days before the delivery were also tested for the dengue NS1 antigen using the Dengue Early ELISA (Panbio diagnostics, Australia) and RT-PCR. Viral RNA was extracted from serum samples using a purification column from the PureLink™ Viral RNA/DNA Kit (Invitrogen, Carlsbad, CA), and conventional one-step and nested PCR [24] was performed to identify the serotype. If the women were identified as recently infected, their neonates were also tested with the dengue NS1 antigen and RT-PCR tests to assess the vertical DENV transmission.

### Past and recent infection definitions

IgG-positive results were considered to indicate previous dengue exposure among parturients or IgG placental transfer in the umbilical cord serum samples. Simultaneously positive results by IgM PanBio and MacElisa and the detection of the dengue NS1 antigen and/or positive RT-PCR results were considered to indicate a recent DENV infection.

### Sample size

A sample of 500 parturients was sufficient to estimate the frequency of ~50% of the dengue infection with 4.5% precision at a 95% confidence interval (95% CI).

### Data analysis

The descriptive data analyses were performed to evaluate the main characteristics of the women and neonates. The prevalence of previous and recent dengue infection and its 95% CI were estimated.

The agreement between the IgG positivity of the matched pairs of parturients and their neonates was calculated using the *Kappa test*. The correlation between the maternal and corresponding umbilical cord serum IgG antibodies indices was calculated (R^2^) and presented in a scatter plot.

The positive and negative predictive values (PPV and NPV) of the self-reporting of past dengue episodes compared with the serologic results were calculated.

All analyses were performed using SPSS 17.0 (SPSS Inc., Chicago, IL) and EpiInfo (CDC 3.5.1 version).

### Ethical issues

This project was approved by the regional Medical Ethics Committee of Humans and Animals of the University Hospital, Federal University of Goias (no 078/2009). All women signed the informed consent regarding their own participation and for the umbilical cord blood specimens and data collection of their offspring. For underage women, the informed consent was signed by their legal guardians.

## Results

**Figure 1:**
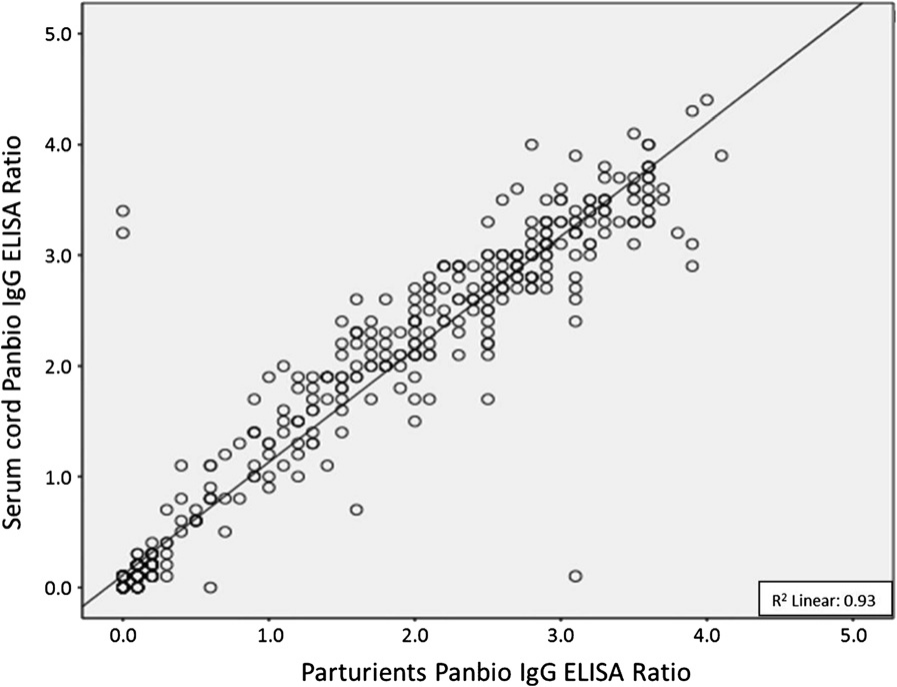
Correlation between maternal and umbilical cord serum of anti-dengue IgG antibodies.

Among the 505 parturients recruited, the prevalence of previous dengue infection was 53.9% (95% CI: 49.4–58.3%). Among new-borns, a 55.0% (95% CI: 50.6–59.4%) IgG seropositivity was detected, yielding 99.3% co-positivity for placental antibody transfer (*Kappa* = 0.96). There was no statistic difference between the positivity of pregnant women and neonates, considering the overlap of the confidence intervals (Table 1). Figure 1 shows the linear correlation between the maternal and neonatal anti-dengue IgG index values (R^2^=0.93).

**Table 1:** **Frequency of past and recent dengue infection among parturients and the umbilical cord sera and the *Kappa *agreement for IgG placental transfer**

**Dengue infection**	**Parturients**	**Cord serum**	***Kappa***
	**(n = 505)**	**(n = 505)**	
Past Infection			
IgG positive (95% CI)	53.9% (49.4–58.3)	55.0% (50.6–59.4)*	0.96
Recent infection			
IgM**positive and RT-PCR detection (95% CI)	2.8% (1.4–4.4)	0%	na

* IgG placental transfer.

**IgM positivity: commercial and in house ELISA tests.

na; not applicable.

A previous history of dengue was reported by 100 pregnant women, yielding 88% (95% CI: 79.6–93.4%) for the positive predictive value (PPV). Of the 405 women with no reported dengue history, 184 were IgG seropositive (45.4%; 95% CI: 40.9–50.2%). A history of yellow fever vaccination was reported by 82.0% of women who had IgG anti-dengue antibodies and by 86.8% without this seromarker of infection.

Overall, 9.3% women reported dengue symptoms or dengue disease during pregnancy, and 1.4% (n = 7) had symptoms in the 10 day period preceding the delivery. For one parturient with recent symptoms at the delivery time DENV2 was detected by RT-PCR but NS1 antigen or IgM antibody were undetectable.

Of the 505 screened parturients, 44 were IgM positive by Panbio, 14 were simultaneously positive by both the commercial and in-house IgM capture-ELISA, and 4 were indeterminate. The frequency of recent infection was 2.8% (95% CI: 1.4–4.4%).

Of the 505 serum cord samples, 4.7% (95% CI: 3.0–6.9%) had IgM anti-dengue antibodies (n = 24) by the commercial kit (Panbio), but no IgM positivity was detected by the in-house ELISA test.

## Discussion

In central Brazil with nearly 15 years of continuous dengue virus circulation, approximately half of the pregnant women screened had detectable dengue antibodies.

Our study showed a high agreement of IgG seropositivity between the pairs of pregnant women and their neonates (99.3%), in agreement with studies from Southeast Asia [25-27]. In our study, there was a positive correlation of the maternal-foetal specific IgG anti-dengue antibody, and even higher levels of the antibody were observed in some offspring samples compared with their mothers. This finding reflects the dynamics of IgG placental transfer, which increases according to the length of gestation and peaks during the last weeks of pregnancy, a well-documented immune process described for several infectious diseases [28].

Reports of clinical dengue symptoms during the gestational period was not common, with most of the parturients being asymptomatic in the short period preceding delivery, even considering that the field study was conducted during the 2010 outbreak. A high ratio of asymptomatic versus symptomatic dengue infection is in agreement with other studies conducted among pregnant women [29] and in the general population [30-32]. Because asymptomatic dengue infections are a common finding, studies aiming to detect the prevalence and incidence of dengue infections among pregnant women and infants are valuable to evaluating the maternal antibody transfer and the dynamics of dengue transmission [10].

Our finding that approximately half of the adult population that was screened was still immunologically naïve to dengue virus exposure appears compatible with the more recent viral re-introduction in central Brazil [31]. There was a lag period of approximately 10 years between the first dengue virus isolation in the Atlantic coast (1985) and its spread toward central part of the country (1994), where this investigation was performed [31]. In a city located in the north-eastern costal region, a population-based serosurvey presented an approximately 90% infection rate among adults in the early 2000’s [30]. Considering that Brazil is a continental country, a large variation in the prevalence of dengue infection has been detected by the serosurveys, based on the study setting and the cumulative period of virus exposure at the population level. Nevertheless, the dengue seroprevalence detected in central Brazil contrasts with the more established dengue circulation of more than five decades in Southeast Asia, in which the adults have mounted immunological responses for all dengue serotypes but the children are the main susceptible group for acquiring dengue infection [27,33].

The incidence of recent infection varied from 2.8% when using commercial test to 8.7% using in-house-test, pointing out differences in sensitivity and specificity of these assays. Approximately 2.0% of IgM seropositivity was detected among parturients in our setting when both anti-dengue virus immunoglobulin M tests (commercial and in-house) were simultaneously positive, according to the recent infection definition of this research protocol. The evaluation of the commercially available anti-dengue virus IgM tests indicates that although the Panbio IgM capture presents a sensitivity and specificity of approximately 80%, varying with the different serotypes and being less accurate among individuals exposed to previous dengue infection [34-36]. Furthermore, the in-house test applied here was formulated/constituted by the Brazilian DENV antigens in circulation, which may confer a higher specificity. As most of the parturients were asymptomatic and the DENV viraemia period is short (~five days), the investigation of recent infection by virological markers was not feasible in this type of cross-sectional design.

Similar results of recent infection around 2% were found among parturients in Southeast Asia during 2006 [29]. The frequency of IgM positivity in both maternal and umbilical cord blood was approximately 8% by commercial ELISA compared with 2.0% in parturients by the in-house test. In the umbilical cord blood, no anti-dengue IgM was detected by the in-house test. The absence of IgM antibodies in the cord serum is in line with the general knowledge that this pentamer immunoglobulin only crosses the placental barrier in rare situations [37].

The apparent discrepancy of the recent infection results, according to the different laboratorial methods employed may be explained by the dynamic of virus exposure and the time sequence of the immune response [38]. For example, IgM antibodies may be detected after the 7 days of onset of symptoms to two to three months after viral exposure in contrast with the short time circulation (days) of the dengue viremia. Therefore, the detection of IgM antibodies in samples collected closely to labor indicate recent dengue infection but participants had undetectable NS1 antigen levels (NS1 Ag) as in our study. NS1 protein assay may be detected in serum samples during viremic period (~five days after the onset of symptoms). Several studies detected relatively low sensitivity of this test, below 50% when samples were collected after seven days of symptoms [39-41]. Another potential reason for the diverse results of viremia by RT-PCR compared to the detection of NS1Ag could be the low sensitivity of NS1 observed among population of Nicaragua and Phillipines. These results were associated with the distinct circulating serotype, duration of illness previous to data collection and the presence of NS1-IgG immune complex among secondary infected individuals with consequently protein clearance [41].

Considering the oncoming dengue vaccine trials, the transfer of dengue maternal antibodies to infants should be taken into account due to the potential reduction of vaccine efficacy and the ideal age of vaccination. Additionally, maternal dengue antibodies are considered to enhance the disease severity among infants [14,15]. Because this study design was cross-sectional, pre- and post-natal follow-up to measure the incidence of dengue infection and clinical dengue cases among women and the kinetics of maternal dengue antibodies among infants were beyond the scope of the current investigation.

One of the limitations of this study was that single or multiple DENV infections by specific subtypes were not identified in the subpopulation of pregnant women and neonates. Detecting the serotypes of DENV by the plaque reduction neutralization test (PRNT) is desirable. However, this laboratory technique is laborious and requires a skilled technical staff [42]. We are also aware of the limited representativeness of the studied population, which included the subpopulation of pregnant women from selected recruitment settings in a city located in central Brazil.

Finally, regional endemic scenarios should be taken into account when modelling the disease burden in this subpopulation and the effectiveness of interventions, such as vaccination, in a large country such as Brazil.

## Conclusion

In central Brazil, the current epidemiological pattern of dengue infection showed that half of the pregnant women had an immunological marker of past dengue exposure with similar frequency of placental transfer of maternal IgG antibodies, providing short-term protection for their neonates. This study provides critical data on the prevalence of transplacental transferred maternal-infant anti-dengue antibodies. The design of future vaccine trials should consider the diverse regional epidemiological scenarios regarding naive and immune population.

## Competing interests

The authors declare that they have no competing interests.

## Authors’ contributions

AFLTA, VCRF, JBSJ and CMTM were in charge for the study design, data analyses, interpretation of the results and writing the manuscript. LAS participated in the study design, laboratory work and the drafting of the manuscript. ACMO participated on the field work and database entry. LAP was in charge for the molecular tests. All authors have read and approved the final manuscript.

## Pre-publication history

The pre-publication history for this paper can be accessed here:

http://www.biomedcentral.com/1471-2334/13/254/prepub
